# *hsphase*: an R package for pedigree reconstruction, detection of recombination events, phasing and imputation of half-sib family groups

**DOI:** 10.1186/1471-2105-15-172

**Published:** 2014-06-07

**Authors:** Mohammad H Ferdosi, Brian P Kinghorn, Julius HJ van der Werf, Seung Hwan Lee, Cedric Gondro

**Affiliations:** 1 The Centre for Genetic Analysis and Applications, School of Environmental and Rural Science, University of New England, Armidale, Australia; 2 Hanwoo Experiment Station, National Institute of Animal Science, RDA, Pyeongchang, Korea

**Keywords:** SNP, Phasing, Imputation, Recombination, Haplotypes, Linkage analysis, Genotyping, Parentage testing, Pedigree reconstruction

## Abstract

**Background:**

Identification of recombination events and which chromosomal segments contributed to an individual is useful for a number of applications in genomic analyses including haplotyping, imputation, signatures of selection, and improved estimates of relationship and probability of identity by descent. Genotypic data on half-sib family groups are widely available in livestock genomics. This structure makes it possible to identify recombination events accurately even with only a few individuals and it lends itself well to a range of applications such as parentage assignment and pedigree verification.

**Results:**

Here we present *hsphase*, an R package that exploits the genetic structure found in half-sib livestock data to identify and count recombination events, impute and phase un-genotyped sires and phase its offspring. The package also allows reconstruction of family groups (pedigree inference), identification of pedigree errors and parentage assignment. Additional functions in the package allow identification of genomic mapping errors, imputation of paternal high density genotypes from low density genotypes, evaluation of phasing results either from *hsphase* or from other phasing programs. Various diagnostic plotting functions permit rapid visual inspection of results and evaluation of datasets.

**Conclusion:**

The *hsphase* package provides a suite of functions for analysis and visualization of genomic structures in half-sib family groups implemented in the widely used R programming environment. Low level functions were implemented in C++ and parallelized to improve performance. *hsphase* was primarily designed for use with high density SNP array data but it is fast enough to run directly on sequence data once they become more widely available. The package is available (GPL 3) from the Comprehensive R Archive Network (CRAN) or from http://www-personal.une.edu.au/~cgondro2/hsphase.htm.

## Background

Identification of recombination events and which chromosomal segments contributed to an individual is useful for a number of applications in genomic analyses including haplotyping, imputation, linkage disequilibrium [1], signatures of selection, and improved estimates of relationship and probability of identity by descent [2]. This is particularly true for genomic prediction which has become an important tool in modern livestock breeding programs to predict the merit of individuals by estimating the genome-wide effects of the alleles they inherited from their ancestors [3]. It is expected that the accuracy of prediction will be even higher once it is based on causal variants identified through sequencing [4] instead of the currently used linked markers. In livestock, individuals of high genetic merit, particularly males, are widely used which leads to an overrepresentation of their genetics across the population. This stratification can be problematic for population based phasing algorithms which rely on samples being unrelated to each other and reasonably representative of the spectrum of genetic diversity [5]. On the other hand this high level of relatedness between individuals provides a structure of high linkage disequilibrium which can be used to track chromosomal segments (haplotypes) throughout the population. By sequencing these overrepresented individuals and genotyping their descendants with high density marker panels, their full sequence data can be imputed [6] for around one tenth of current sequencing costs. Availability of sequence data for a large number of samples will increase the power to identify causal variants, which in turn can replace the currently used evenly spaced marker panels with a smaller subset of trait specific variants that are either causal or in perfect LD with the causal variants [4]. This implies the ability to accurately identify and track haplotypes in the population.

Here we present *hsphase*, an R package that implements a fast, deterministic and robust method for half-sib family structures to identify recombination events, phase family groups, impute and phase un-genotyped sires and build a library of haplotypes [7]. The package also makes use of this population structure to evaluate correctness of recorded pedigrees, identify and fix pedigree errors, i.e. reassign individuals with wrong pedigree records to their correct sires; or even reconstruct family groups without pedigree records. If genotypes from candidate parents are available the package can be used for parentage verification.

Additional functions allow identification of genomic mapping errors, evaluation of phasing results generated by *hsphase* or other phasing programs. *hsphase* will also generate a *blocking structure* of chromosomal segments that define which progeny carry segments identical by descent. This can be used to improve phasing of the paternal sequence data [8,9] and allows precise sequence imputation in the offspring. Imputation is important in association studies and genomic prediction to increase accuracy and power since a large number of samples can be genotyped at lower density (and lower cost) and imputed up to sequence level or to denser marker panels, which increases the level of linkage disequilibrium between SNP and causal variants [10].

*hsphase* seamlessly integrates into the R environment for pipelined analyses and provides a range of diagnostic plotting functions that permit rapid visual inspection of results and evaluation of datasets. Functions for pedigree checking, reconstruction and parentage assignment can be used independently or as part of phasing workflow. For phasing purposes, the main advantages of *hsphase* are that is it extremely fast in comparison to population based phasing methods, can be used with small datasets and it is not affected by sampling stratification. It also builds blocks of chromosomal inheritance in the half-sibs which makes it simple to impute when paternal sequence or higher density marker haplotypes are available. The package is sufficiently fast to be used directly on sequence data.

## Implementation

The *hsphase* package exploits the linkage disequilibrium found within a half-sib family and the information content of opposing homozygous SNP markers [11]. An opposing homozygote, for any given marker, is defined as one individual being homozygous for an allelic variant and the other individual homozygous for the alternative allele.

**Figure 1:**
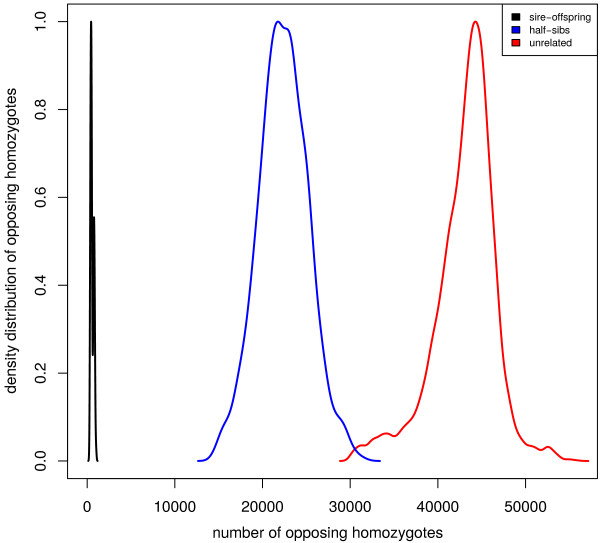
**Density plot of opposing homozygotes.** The three distributions show the number of opposing homozygotes between parents and offspring (black), between half-sib families (blue) and between unrelated individuals (red). The distributions are genome wide and based on 290 Hanwoo cattle from 36 sires genotyped on the 700 k Illumina BeadChip. The separation between parent-offspring and other relationships is very clear but there is some level of overlap between the distributions of half-sibs and unrelated animals which can make it not possible to perfectly characterize family groups. The level of separability is conditional on the overall genetic variation in the population and is better with genetically diverse populations. For reference purposes, Hanwoo cattle have small effective population size (~100) and are subject to some ascertainment bias in the array design which further constrains detectable variation.

Consider, for example, a sire-offspring relationship, opposing homozygotes can be used to identify Mendelian inconsistencies which should not occur in a true relationship apart for genotyping errors or an unlikely mutation. Alternatively, between unrelated individuals the number of opposing homozygotes is much higher. This difference can be used to e.g. exclude a parentage relationship [11]. The same applies to other relationship levels, with half-sibs showing less opposing homozygotes between themselves than unrelated individuals (Figure 1). While the relationship between parent-offspring is essentially 100% accurate with a high enough number of markers, the separation between half-sib groups and unrelated individuals is not always so clear cut (Figure 1). However the two distributions are still highly separable and can be used to assign relationships. *hsphase* implements four different methods to define the separability between related and unrelated individuals. The first method uses a pre-determined cut off based on the maximum number of expected opposing homozygotes in a family. The second approach uses the regression coefficients (slope and intercept) estimated from a large population of sheep half-sib families genotyped on the Ovine50k Illumina BeadChip; these are the default values based on a simple linear regression of the number of opposing homozygotes, but user defined coefficients better suited for a particular population can also be used. The third approach implements the method proposed by Calus e*t al.*[12] based on the expectation of opposing homozygous loci in half-sib families in contrast to unrelated individuals using population wide allele frequencies. The cut off value used to accept a relationship is 90% of the average difference between the predicted number of opposing homozygotes in unrelated individuals and half-sib families. The last method uses the expected number of recombinations to define relationships; *hsphase* uses opposing homozygotes to build blocks of haplotypic relationships in family groups, if an individual is not truly part of a group it will need a large number of recombinations to maintain Mendelian consistency with the other family members. These recombinations are of course not real but can be used to exclude a relationship (further details in the *block structure* section). To build the pedigree itself, the matrix of opposing homozygotes is used to calculate the Manhattan distance between all pairs of individuals. The algorithm then recursively builds a distance matrix and hierarchically clusters them into two groups using Ward’s minimum variance method. At each iteration, the groupings are checked against the separation criterion being used and if all individuals are below the threshold they are assigned to a family group; else the process is repeated.

**Figure 2:**
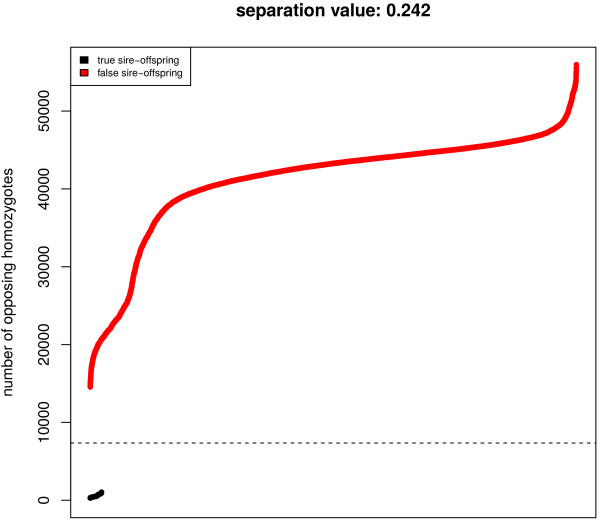
**Separation value between true and false parent-offspring relations.** The separation value is the difference between the smallest number of opposing homozygotes found across all false sire-offspring relations (i.e. all pairwise combinations except the real sire-offspring pairs) and the maximum number of opposing homozygotes in the correct sire-offspring pairs divided by the maximum number of opposing homozygotes found in the dataset. Positive values allow reliable identification and exclusion of parent-offspring relationships. The figure shows sorted pair-wise combinations of opposing homozygotes for 326 Hanwoo cattle genotyped on the Illumina 700 k BeadChip (290 offspring and their 36 sires); true sire-offspring relations in black and false in red. The jagged line shows a cut off threshold of 1% genotyping errors.

For parentage assignment a square matrix with the number of opposing homozygotes between all pairs of individuals is calculated. Parent-offspring pairs can then be assigned based on a maximum number of allowable Mendelian inconsistencies (e.g. 1% genotyping error). Figure 2 illustrates for a cut off threshold of 1% genotyping errors. For small parentage panels (~100-200 SNP) the maximum number of mismatches allowed should be between 0 and 2. If genotypes from a known pedigree are available they can be used to calculate the *separation value* which is the difference between the smallest number of opposing homozygotes found across all false sire-offspring relations (i.e. all pairwise combinations except the real sire-offspring pairs) and the maximum number of opposing homozygotes in the correct sire-offspring pairs divided by the maximum number of opposing homozygotes found in the dataset (Figure 2). The separation value can be useful to design parentage panels or test their efficacy for parentage testing. The higher the value, the better the panel is at resolving parentage assignments and, if the value becomes zero or negative, a perfect separation between true and false sire-offspring relations is not possible.

**Figure 3:**
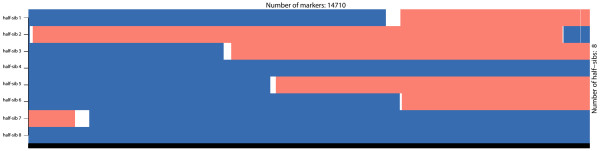
**Block structure for a half-sib family.** The block structure shown is for chromosome 29 of eight half-sib Hanwoo cattle genotyped on the 700 k Illumina BeadChip array. The two colours indicate the paternal and maternal strands from the paternal line each individual inherited (i.e. the individual’s relationship with the two haplotypes of the sire); white regions are areas where the phase could not be determined.

Opposing homozygotes are also used for phasing and *hsphase* implements the method described in [7]. Briefly, all opposing homozygous loci are identified and these unambiguously identify heterozygous sites in the parent. These are then used to assort individuals into groups according to the paternal allele they received, which in turn provides information about the most likely phase in the ancestor. Blocks of consecutive markers that are co-inherited across groups become obvious at this stage and allow detection of recombination points. These can be visualized as a *block like structure* across the individuals’ genome where each block reflects the inherited paternal haplotype (Figure 3). Paternal haplotypes are inferred by simply averaging the sum of the genotypes at each marker that inherited a particular strand (block) from its ancestor. Paternal haplotypes in the offspring are identified by matching the inherited blocks in the offspring with the phased haplotypes of the parent. Maternal haplotypes are then obtained by subtracting the paternal haplotype from the individual’s genotype. The method does not depend on population parameters and even small datasets give accurate results (R^2^ > 0.9 for block detection and imputation for families > = 8 individuals). A detailed description of the algorithm implemented in *hsphase* and an evaluation of its performance is given in [7]. To improve the algorithm’s performance most of the code was written in C++ and parallelized.

*hsphase* was implemented as a package for the widely used R statistical programming environment and wrapper functions make it easy to use and facilitate integration with other R/Bioconductor packages. Programs such as *snpQC*[13] output files in a format that can be used by *hsphase*. Source code, compiled package, tutorial and example dataset are available from the project’s website (the package is also available directly from CRAN). In the following section, the main components of the package are briefly described.

## Main functions in *hsphase*

### Input data

*hsphase* requires a SNP map file (name, chromosome and map positions), a genotype data file (numerically coded as 0, 1, 2 for the three genotypes and 9 for missing data) and a pedigree file (individuals and paternal ancestor). The latter can be generated from the data itself if no pedigree information is available or the pedigree is unreliable.

### Pedigree reconstruction and parentage assignment

**Figure 4:**
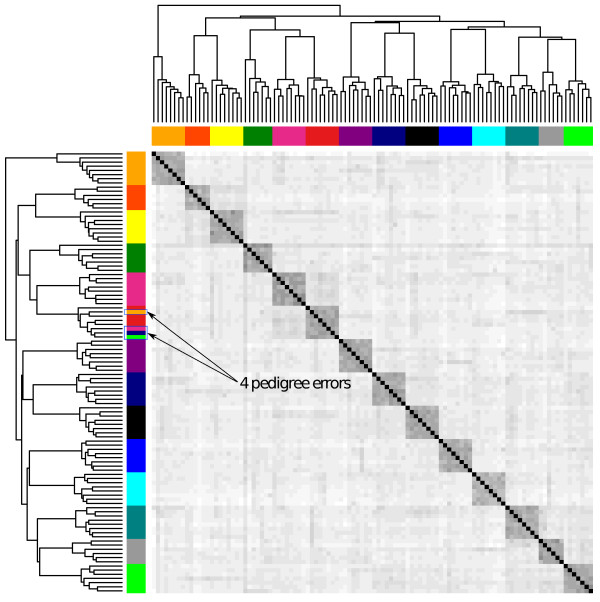
**Correction of pedigree errors.** The heatmap shows the relationships between individuals based on opposing homozygotes. Half-sib families are colour coded. On the vertical bar (left-hand side), 4 individuals are misclassified in the phenotypic pedigree. The horizontal bar (top) shows the reconstructed pedigree using the *rpoh* function in hsphase. Data is for 106 Hanwoo cattle from 14 family groups genotyped on the 700 k Illumina BeadChip array. Four pedigree records were purposely swapped. The darker blocks on the diagonal help identify half-sib groups.

The function *ohg* calculates a square matrix with the number of opposing homozygotes between all pairs of individuals; these are then used by *rpoh* to reconstruct family groups by iterative hierarchical clustering in the absence of pedigree data. If a pedigree file is available the function *pedigreeNaming* will match the inferred family groups with the most likely parents and can be used to correct pedigree errors or to evaluate efficiency of pedigree reconstruction. The accuracy of pedigree assignment decreases if individuals belong to overlapping generations with common ancestors. Results of pedigree reconstruction can be visualized with the *hh* function which generates a heatmap of the relationships for easy detection of pedigree errors in the original pedigree (if available) and evaluation of pedigree assignment (Figure 4). The *rpoh* function calculates a distance matrix from the opposing homozygotes matrix and was written in C++ with multithreaded support to accommodate large datasets.

**Figure 5:**
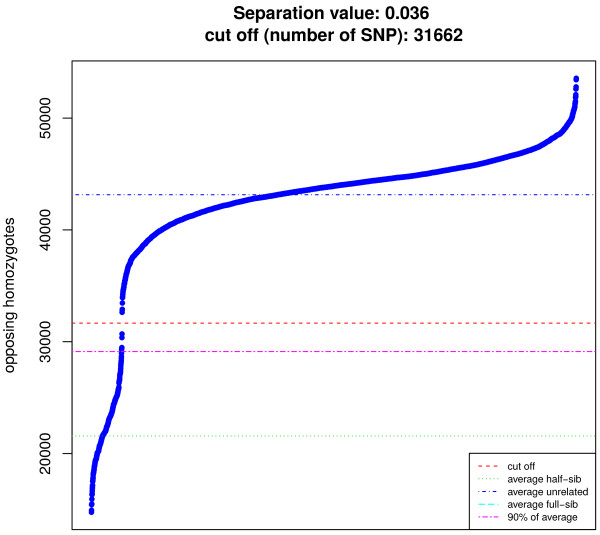
**Sorted pairwise numbers of opposing homozygotes with *ohplot.*** The plot is useful to guide decisions for parameter settings to reconstruct the pedigree. The *separation value* is the maximum separation found between sorted value pairs divided by the maximum number of opposing homozygotes. The *cut off* value is the number of SNP at the mid-point of the largest separation (red line). The other lines are the average number of opposing homozygotes expected in full-sib families (cyan – not shown here), half-sib families (green) and in unrelated individuals (blue) according to [12]; plus the 90% threshold used for pedigree reconstruction (pink). The function can also be used with pedigree information to detect inconsistencies (relations are colour coded according to the pedigree to facilitate visualization). The plot is from 106 Hanwoo cattle from 14 family groups genotyped on the 700 k Illumina BeadChip array.

These functions are not restricted to dense marker panels and can be used to assign parents using small parentage testing panels, for example. Function *pogc* takes the matrix of opposing homozygotes as input and returns a pedigree of parent-offspring assignments based on the maximum number of mismatches allowed (user defined parameter – default 1% of the number of markers). Function *ohplot* also uses the matrix of opposing homozygotes and a pedigree file to plot the separation value of the dataset and the sorted results of all pairwise comparisons (Figure 5). *ohplot* is useful to check pedigrees, evaluate parentage testing panels and guide decisions on acceptable mismatch thresholds. The function can also be used without pedigree information and will simply sort and plot the values of the upper triangle of the opposing homozygotes matrix, the separation value reported is then the maximum separation found between sorted value pairs. It can help to identify the maximum number of opposing homozygotes allowed for pedigree reconstruction (see results section). The function also reports average values for the number of opposing homozygotes expected in full and half-sib families and in unrelated individuals according to [12]; plus the 90% threshold used for pedigree reconstruction.

### Block structure and recombination events

The *bmh* function creates the blocking structure for the half-sibs and splits them into two groups based on the chromosomal segments they inherited from either one of the sire’s haplotypes. Blocks for each chromosome are constructed by selecting the first opposing homozygous SNP on the chromosome and partitioning all members of a half-sib family into two groups according to their genotypes (i.e. all individuals with genotype *AA* are placed in one group – *group 1*, and all with *BB* in the other group – *group 2*). Starting from this initial grouping the function steps through the SNP according to their map order to allocate individuals into one group or the other one, until the end of the chromosome is reached. At the end of the process each individual at each SNP will have been assigned to one of the two groups; the function returns a matrix of individuals by SNP coded as *1* and *2*. Recombination between two adjacent SNP is an unlikely event, so from the second SNP onwards individuals are assigned to a group by minimizing the number of individuals that have to change groups in relation to the previous grouping (i.e. minimum number of recombinations). Recombinations are identified when an individual moves from one group to the other based on its opposing homozygous status. The *bmh* function performs a validation step for the recombination by checking if during the next steps (SNP) the individual does not return to the previous group. Recombinations occurring on both sides of a single SNP in a single individual are interpreted as a genotyping error and ignored. Group assignment is based on family relationships which makes *bmh* sensitive to pedigree errors [14]. In addition, only a proportion of SNP will be homozygous for any given individual at any particular SNP; family sizes need to be sufficiently large to be able to reliably assign individuals to groups and markers sufficiently dense to correctly detect recombination events. As rule of thumb, families with at least 8 individuals and 50 k panels should yield very accurate results.

**Figure 6:**
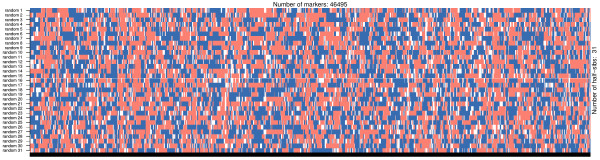
**Phasing error detection using blocks.** The two colours indicate the paternal and maternal strands from the paternal line each individual inherited. The large number of recombinations and unresolved areas (in white) is indicative of poor phasing results. The figure was simulated by using 31 random and unrelated individuals (bovine chromosome one).

If parent-offspring haplotypes are available, the *hbp* function can be used to compare an offspring’s haplotypes with its ancestor’s haplotypes. The function returns a matrix with the same block structure of *bmh* and can be used to evaluate phasing results of other programs – a single parent-offspring is sufficient in this case. Combined with the *imageplot* function (plots the block structures – Figure 6) it is straightforward to evaluate results and identify problems. Extreme recombination patterns on the *imageplot* are indicative of incorrect phasing. If individuals are unrelated the *imageplot* will have a chaotic structure (Figure 6) which can be also used to check the pedigree.

**Figure 7:**
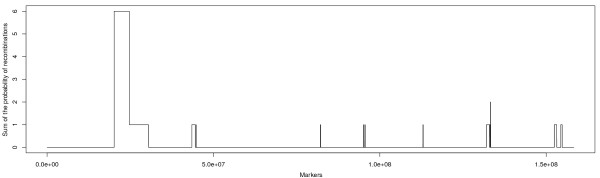
**Distribution of recombination events.** The plot shows the probability of recombination along chromosome one (46495 SNP) for 106 Hanwoo cattle; this is useful to identify local variation in recombination rates. In this case the population size is too small to reliably detect these regions. Note however the high value in the first part of the chromosome, these are not true recombinations but mapping errors instead, probably due to an assembly error in the reference sequence.

The function *pm* uses the block matrix as input and returns a matrix of all recombination events per individual and between SNP. Function *recombinations* returns a count of the number of recombinations per individual. *rplot* displays recombination counts per SNP and assists identification of local variation in recombination rates or mapping errors (Figure 7).

### Phasing and imputation

The function *ssp* imputes and phases the paternal haplotypes. The function infers the sires’ haplotypes at each SNP by simply averaging the sum of the genotypes of the half-sibs in a blocking group (alleles coded as 0 and 1; genotypes as 0 – 0/0, 1 – 0/1 and 2 – 1/1). Averages are rounded to the nearest integer and assigned to the sire’s haplotypes.

The *phf* function phases the offspring and returns their paternal haplotypes. It uses the sire’s phased haplotypes as a reference and overlaps the block matrix to select which parts of the haplotypes each individual inherited. Once the paternal haplotypes of the offspring are created, the maternal ones are obtained by simply subtracting these haplotypes from the original genotypes.

The function *impute* imputes the paternal strand of half-sib families from low density genotypes to high density by using the sire’s haplotypes as a scaffold. Similarly to the function *phf* it simply uses the blocks to match the haplotypes of the offspring with the correct haplotype of the sire and fills the missing markers with the haplotypes of the denser panel.

For large datasets the *para* function provides a parallelized wrapper to partition the job across multiple CPUs.

## Results

To discuss the use of the *hsphase* package, a dataset of 106 brown Hanwoo Korean cattle genotyped on the Illumina 700 k BovineHD BeadChip SNP array was used. Individuals belonged to 14 half-sib family groups with family sizes ranging from 6 to 8. Genotypes for the 14 sires were also available and pedigree records were accurate. For reference purposes the Korean Hanwoo are a pure-bred heavily selected population with a small effective population size (Ne ~100) and there is some ascertainment bias in the chip which was not specifically designed for the breed. Population differences among unrelated individuals is expected to be lower than in populations with large Ne.

### Pedigree reconstruction

**Figure 8:**
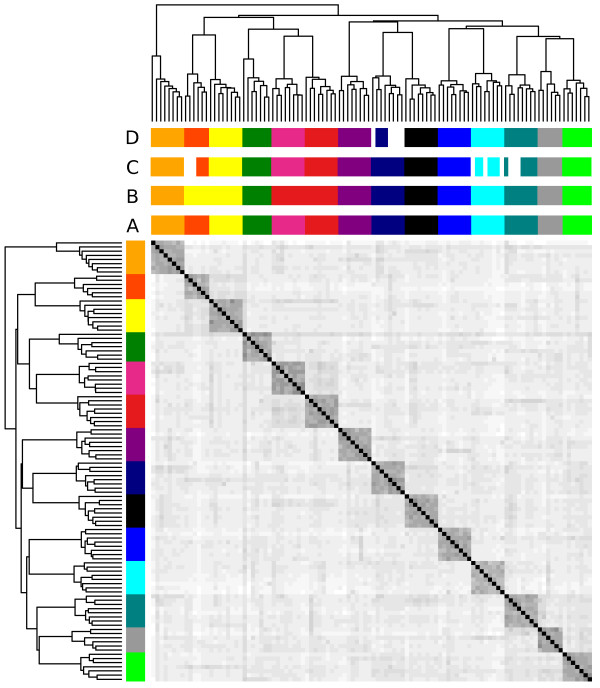
**Comparison of pedigree reconstruction methods.** Left-hand side colour bars for true family relationships, top-side bars for inferred pedigree and pedigree matching. The top colour bars are respectively, A) Manually determined threshold for number of opposing homozygotes in a half-sib family (31,662). The pedigree is 100% accurate and correctly assigned to the sires. B) Regression coefficients derived from a large sheep population. Two families did not separate (yellow and red – top bar) and were classified as a two groups instead of four. C) The third method based on [12] failed to assign 9 individuals to their correct sire (unassigned individuals shown in white). Method four uses number of recombinations to sort family groups. Here chromosome one was used with a maximum of 10 recombinations allowed. The method failed to group 5 individuals from one family (unassigned individuals shown in white).

As previously discussed *hsphase* implements four methods to define thresholds for pedigree reconstruction. Unfortunately no approach is robust across any scenario and the different methods will perform better or worse in different situations. The accuracy of reconstruction will depend on the sample size and genetic diversity in the population. A first look at the data using *ohplot* (Figure 5) can provide some indications as to the separability of family groups from unrelated individuals and help define appropriate parameters for the different methods. For this data there is a clear separation between half-sibs and unrelated individuals but this is not always the case, as shown in Figure 1. Due to the clear separability of the dataset, the first method (manually defining a maximum number of allowable opposing homozygotes in a family) was able to perfectly reconstruct the pedigree (Figure 8, colour bar A) using the cut off value shown in Figure 5 (31,662). The second method (Figure 8, colour bar B) using regression coefficients derived from sheep was unable to split two family groups; i.e. four families were grouped into two. There was no family mixing which suggests that the coefficients are too stringent for this population and probably suboptimal. A wider tolerance would have allowed the final split. We have obtained good results with sheep populations and other cattle breeds using these coefficients (results not shown) but this approach may lack generalization. The third method follows [12] (Figure 8, colour bar C) and failed to assign 9 individuals to their correct sires (92% correct). Albeit not perfect, the accuracy is still high and in other scenarios it works well; when we used this approach on the full Hanwoo data (36 sires, 290 offspring) we obtained 100% accurate pedigree inference (data not shown). The lower accuracy here is probably due to the low numbers of individuals and imprecise allelic frequency estimates which suggests the method is better suited for large sample sizes or when population allelic frequencies are available from another source. The last method uses the blocking structures within families to count recombination events and builds groups based on a maximum allowable number of recombinations. Figure 8 (colour bar D) shows the reconstructed pedigree using chromosome one as a reference with a maximum of 10 recombinations allowed. The method failed to group 5 individuals from one family (95% correct). This approach requires some prior knowledge of recombination expectations per chromosome. If the number of recombinations allowed was increased to 14 the pedigree would be perfect (same as Figure 8, colour bar A). Other chromosomes can be used (e.g. chromosome 29 with 4 recombinations is 100% accurate). As a rule of thumb, shorter chromosomes with less recombination tend to yield better results.

**Figure 9:**
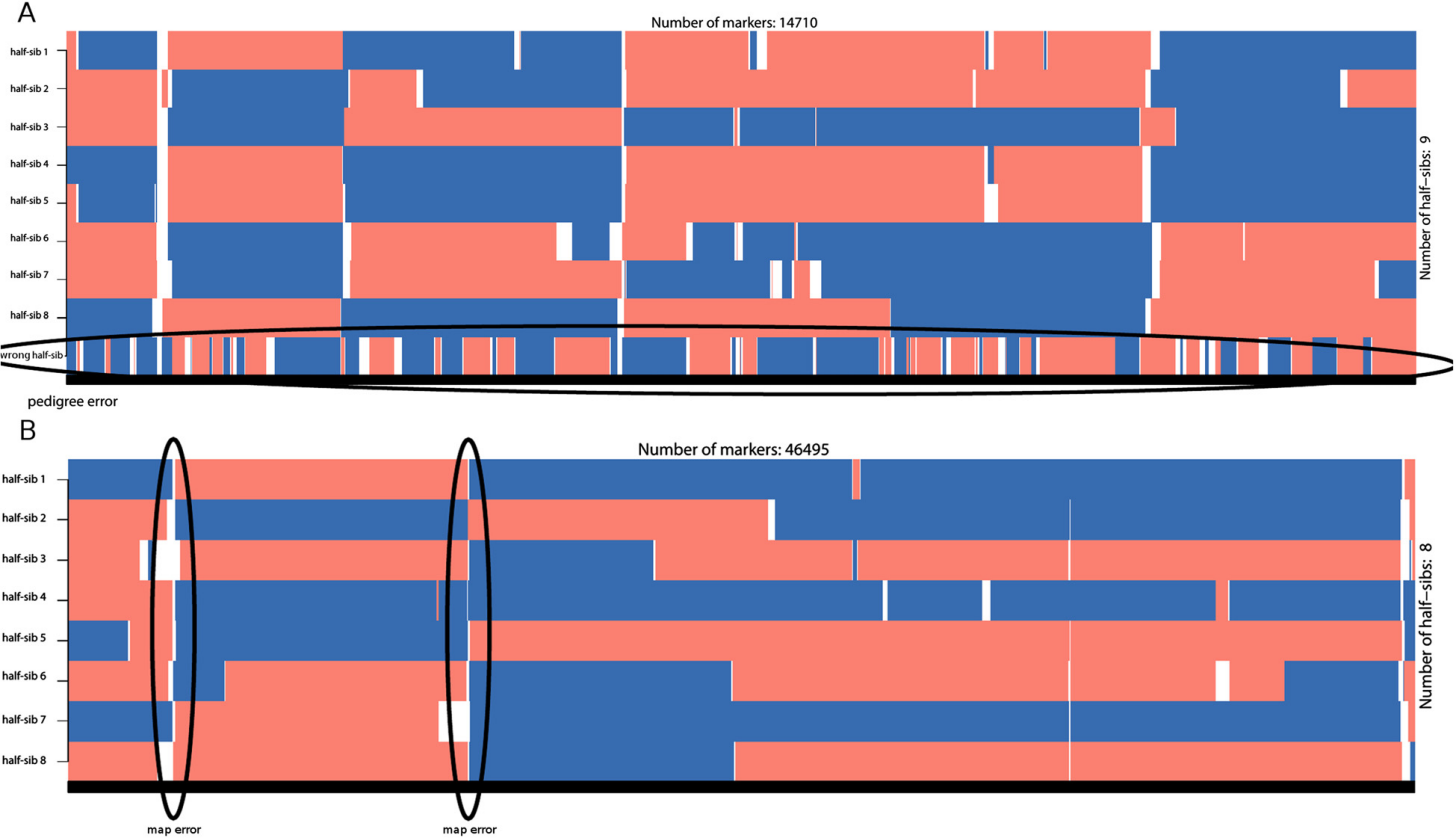
**Error detection using blocks.** Plots generated using the *imageplot* function. **A)** The last individual is wrongly assigned to the family group (excessive number of short recombinations). Note that the inclusion of a wrong individual causes problems with block assignment for the whole family (compare the block structure for the same group in Figure 3). Data shown is for bovine chromosome 29 with 8 half-sib and an unrelated individual intentionally included. **B)** Putative map error on chromosome 1. A map error has a cumulative effect and causes problems downstream evidenced by chromosome blocks consistently breaking across all individuals.

Generally speaking all methods are quite susceptible to the parameters used. Some care should be taken to define adequate ones for the data at hand and check if the results seem satisfactory (the blocks across the diagonals of the heatmap can be useful to visually identify the families – Figure 8). Another *sanity* check is to inspect the block structures of the families which should not exhibit excessive numbers of recombinations (Figure 9A).

### Map errors

Map errors due to errors in the reference assembly can also be identified by visual inspection of the block structures (Figure 9B). This is characterized by an individual SNP (or a few SNP in a region) that shows an excessive number of recombinations. Map errors are consistent across families, meaning that the same SNP show excessive recombination across all family groups. With the method used in *hsphase*, a map error leads to downstream blocking problems and individuals start showing patterns of recombination at the same SNP (Figures 7 and 9B). This can be corrected by deleting the region with the map error, provided it is not too long. The difference between map errors, regions of high recombination and SNP genotyping problems are not entirely straightforward, particularly if the marker panel is not very dense.

### Accuracy of sire inference and imputation

To test the accuracy of imputation from low to high marker density we selected 46,174 SNP – the SNP in common with the 50 k bovine panel – in the Hanwoo offspring and excluded the others. We built the block structures for this subset of SNP and then used the *impute* function to fill the gaps using the sire’s phased genotypes as a scaffold. The average accuracy of imputation (proportion of paternal haplotypes correct out of total) for the 106 offspring was 0.981 (comparison of 50 K imputed to 700 k with the true 700 k haplotypes). The worst accuracy was 0.977 and the best 0.993. Note that the accuracies were high but they were probably biased upwards since the sires were phased using *hsphase* and there is some circularity in these values. In the absence of *true* phased sire data this issue cannot be resolved unambiguously. We also evaluated the accuracy of sire inference (comparison of inferred genotypes with the true genotypes of the sires). The average accuracy was 0.992, with the worst sire 0.985 and the best 0.997. Undefined regions were not called (average 16.5% of SNP). A comprehensive evaluation of the phasing method used in *hsphase* is given in [7].

### Recombination events

**Figure 10:**
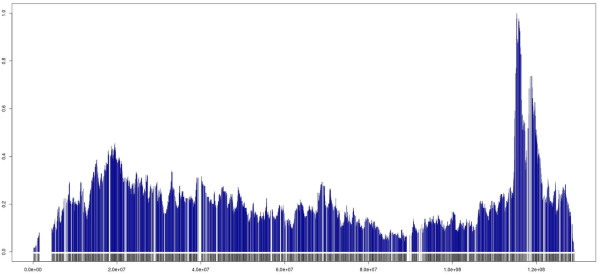
**Recombination pattern on chromosome 6 in sheep.** The figure shows regions of high and low recombination in a large sheep population genotyped on the Ovine 50 k Illumina BeadChip. White areas are regions without SNP coverage on the array. At the end of the chromosome there is a region with over twice the average number of recombinations.

The Hanwoo data is too small to reliably identify local variation in recombination rates (Figure 7). Instead, for illustration purposes, we used a large population of sheep (14005 individuals in 343 families) genotyped on the Illumina Ovine 50 k BeadChip (Figure 10 illustrates recombination patterns on chromosome 6). For large datasets analyses can be sped up with the *para* function which allows jobs to run in parallel and acts as a wrapper for the main functions in *hsphase*. Although this population was reasonably large the time needed for the analysis without parallelization was ~55 seconds and with 8 cores this came down to ~28 seconds. The main reason for the nonlinear speed increase is that each individual analysis was fast and there is limited benefit from the parallelization (too much time is spent on communication across nodes [15]). Speed increases are more obvious with denser marker panels (e.g. sequence data).

## Conclusion

*hsphase* is an R package for analysis and visualization of genomic structures in small half-sib groups. The package can be used to reconstruct pedigree, assign or verify parentage, impute and phase un-genotyped paternal ancestors, phase the half-sib groups and detect and quantify recombination events. Diagnostic plots assist identification of pedigree, mapping and phasing errors. Whilst designed for high density SNP arrays the algorithm is extremely fast and can be used directly on sequence data as it becomes available. Auxiliary functions to impute from low to high density markers and parse datasets are also included in the package.

## Availability and requirements

The package is freely available (GPL 3) from the Comprehensive R Archive Network (CRAN) or from http://www-personal.une.edu.au/~cgondro2/hsphase.htm. Source code, compiled package, a tutorial and example dataset are available from the project’s website.

•**Project name:** hsphase

•**Project home page:**http://www-personal.une.edu.au/~cgondro2/hsphase.htm

•**Operating system(s):** platform independent

•**Programming language:** R [16] and C/C++

•**Other requirements:** the package depends on the R packages *snowfall*[17], *Rcpp*[18,19] and *RcppArmadillo*[20].

•**License:** GNU GPL 3

## Competing interests

The authors declare that they have no competing interests.

## Authors’ contributions

MF and CG designed the algorithm and experiments to test it. MF wrote the R package. SHL, JHJW and BPK advised on the experimental design to test the package. All authors read and approved the final manuscript.
